# Long-term outcomes of liver transplantation for biliary atresia and results of policy changes: over 20 years of follow-up experience

**DOI:** 10.3389/fped.2023.1242009

**Published:** 2024-03-01

**Authors:** Yiyoung Kwon, Yoon Ji Ahn, Jaehun Yang, Eun Sil Kim, Yon Ho Choe, Sanghoon Lee, Mi Jin Kim

**Affiliations:** ^1^ Department of Pediatrics, Inha University Hospital, Inha University School of Medicine, Incheon, Republic of Korea; ^2^ Department of Pediatrics, Samsung Medical Center, Sungkyunkwan University School of Medicine, Seoul, Republic of Korea; ^3^ Department of Surgery, Gil Medical Center, Gachon University School of Medicine, Incheon, Republic of Korea; ^4^ Department of Pediatrics, Kangbuk Samsung Medical Center, Sungkyunkwan University School of Medicine, Seoul, Republic of Korea; ^5^ Department of Surgery, Samsung Medical Center, Sungkyunkwan University School of Medicine, Seoul, Republic of Korea

**Keywords:** biliary atresia, liver transplantation, graft failure, complication, risk factor

## Abstract

**Objective:**

Biliary atresia (BA) patients develop chronic liver disease after the Kasai operation and are eventually indicated for liver transplantation (LT). The purposes of this study were to analyze long-term outcomes after LT and risk factors that affect complications to reduce graft failure.

**Study design:**

Overall, 145 pediatric patients who underwent LT between June 1996 and June 2020 after a diagnosis of BA were included. We performed a retrospective analysis of medical records and evaluated patient and graft survival, cumulative incidence of complications, risk factors, and the results of policy changes.

**Results:**

Patient and graft survival rates in over 20 years were 95.8% and 91.0%, respectively. Post-transplantation lymphoproliferative disease was frequently observed in the early period of immunosuppression within the first 1–2 years after LT. The incidence of cholangitis and rejection steadily increased over time. Weight-to-portal vein size was evaluated as a risk factor for cholangitis and bile duct strictures (OR = 12.82, *p* = 0.006 and OR = 16.54, *p* = 0.015, respectively). When evaluated using 2013 as a reference point, the split graft indication was expanded and the group that received LT after 2013 had a significantly lower survival over time compared with that of the group that received LT before 2013 (*p* = 0.006).

**Conclusion:**

This study revealed time differences in prevalence of complications. The evaluation of weight-to-duct or vessel size is a more important factor in considering complications than the graft-to-recipient weight ratio. Survival outcomes may have been altered by a policy change that affects the donor type ratio in transplantation.

## Introduction

Biliary atresia (BA) is a progressive inflammatory obstructive disease of both intrahepatic and extrahepatic bile ducts. This congenital disease occurs in approximately 1 in 10,000 births in East Asia and causes secondary biliary cirrhosis (1). If the disease is left untreated, it could lead to death within two years, with a median survival duration of 8 months. Although the Kasai operation has been accepted as 1st line treatment for improving the survival rate of patients with BA, 66% of patients develop chronic liver disease after surgery and are eventually indicated for liver transplantation (LT) (2). Hence, BA is a representative disease group that requires LT in pediatric patients, which accounts for at least 50% of all cases.

Owing to LT, the survival rate of BA has increased to above 90%; however, the graft survival rate is lower than the patient survival rate (3). Since patients with BA undergo LT at a young age, several complications occur during the long-term follow-up period. Owing to these complications in the transplanted liver, a chronic liver disease develops and eventually leads to graft failure that requires re-LT. These complications include recurrent cholangitis, acute or chronic rejection, post-transplantation lymphoproliferative disease (PTLD), bile duct stricture, portal vein stenosis, and hepatic artery or vein stenosis (4–6).

Transplantation performance can be affected by the transplantation policies. South Korea expanded the indications for LT of split grafts in deceased donors in 2013 to address the high demand for LT and the insufficient supply of donor livers (7). As a result, the number of patients with BA who underwent split-graft transplantation also increased. The difference in graft survival according to donor type has already been clarified (8, 9); however, if the survival rate is affected by a policy change as a starting point, the policy change can also have great significance.

Beyond survival rates and relatively simple survival-related factor analysis, this study took advantage of LT at a young age to examine the long-term complication rate in patients with BA who underwent LT and to evaluate complications that are related to graft failure. In addition, the pre- and intra-operative factors associated with complications related to the bile ducts and vessels were evaluated. Finally, this study attempted to evaluate the survival rate in relation to donors after the 2013 policy to increase the indications for split grafts of deceased donors.

## Methods

### Patients and study design

This study retrospectively enrolled patients from a single tertiary referral center in South Korea. Patients' medical records from June 1996 to June 2020 were reviewed, and 145 pediatric patients who underwent LT after a diagnosis of BA was made were enrolled in this study. The Kasai operation was the treatment strategy considered in all cases when BA was first diagnosed. However, LT was performed without performing the Kasai operation in some patients who had already developed complications related to decompensated cirrhosis and portal hypertension at the time of diagnosis. LT was also performed in patients in whom decompensated liver cirrhosis and portal hypertension progressed after the Kasai operation. Additionally, re-LT was performed in patients who were evaluated for graft failure after transplantation.

The primary objective of this study was to examine the long-term complication rate in patients with BA who underwent LT, and to evaluate the complications that were directly related to graft failure. The second objective of this study was to evaluate the survival rate in relation to donors after the 2013 policy to increase the indications for split grafts of deceased donors. All study procedures were performed in accordance with the relevant guidelines and regulations and were approved by the Clinical Research Ethics Committee of the Samsung Medical Center (IRB file no. SMC 2021-12-062).

### Data collection

The clinical data of the patients included sex, body weight, age at the time of diagnosis of biliary atresia, age at the time of LT, and donor graft type. Pre-operative evaluations included the pediatric end-stage liver disease (PELD) score; laboratory results including liver function enzymes, bilirubin, albumin, prothrombin time (INR), and ammonia level; and imaging data with ultrasonography, computed tomography, or magnetic resonance imaging. Intraoperative evaluations included the graft-to-recipient weight ratio (GRWR), ischemic time, hepatic vein size, portal vein size, and bile duct size of the grafts. The sizes of each vessel and duct were measured with a ruler and recorded data were collected. When performing statistical analyses, the size (mm) of vessels and bile duct of donor were divided by the body weight (kg) of recipient and converted to size per body weight. Post-operative complications, such as cholangitis, rejection, PTLD, portal vein stenosis, and bile duct stricture, were based on medical records and a review of treatment at the time of the complications. The time frame as a variable was formed with the date on which complications occurred after transplantation was confirmed.

### Surgical methods and postoperative care

During the Kasai operation, the surgeon removed a structure, presumed to be the common bile duct from the gall bladder, exfoliated it, and performed suture ligation. The surgeon again dissected along the structure presumed to be the hepatic duct, and excised it after reaching the liver parenchyma. The surrounding hepatic artery was preserved to prevent injury. After transection of the jejunum about 20 cm below the Treitz ligament, a retro-colic elevation of about 50 cm was performed and portoenterostomy was performed followed by end-to-side jejunojejunostomy. After the Kasi operation, all patients were administered medical prophylaxis of sulfamethoxazole/trimethoprim and phenobarbital for 6 months. Prednisolone also was started with a dose of 5 mg/kg at postoperative day 7, which was tapered within 1 to 2 weeks. Supportive medications, such as ursodeoxycholic acid and multivitamins, were also administered.

In LT with a living donor, the donor's left lateral section was used in most cases. The hepatic vein and artery were anastomosed continuously. The main portal vein to left portal vein anastomosis was performed with sufficient growth factors to prevent anastomotic strictures. For the bile duct construction, hepaticojejunostomy was performed by connecting the jejunum with the Glisson's capsule of the graft liver with stent insertion. An *in situ* split technique similar to that used in living donors was usually performed in the case of deceased donors. After the LT, prophylactic antibiotics (cefotaxime and ampicillin/sulbactam) were administered. In high-risk groups for CMV or EBV, ganciclovir at a dose of 5 mg/kg was also used. After immunosuppressants (IS) were administered, itraconazole was added. Two or 3-drug regimen with calcineurin inhibitor (CI), steroids, mycophenolate mofetil (MMF) was used for IS. In an effort to reduce the use of IS, routine MMF has not been used since the mid-2010s, and CI and MMF were monitored by concentration evaluation to determine whether they were within the therapeutic range.

### Statistical analysis

**Figure 1:**
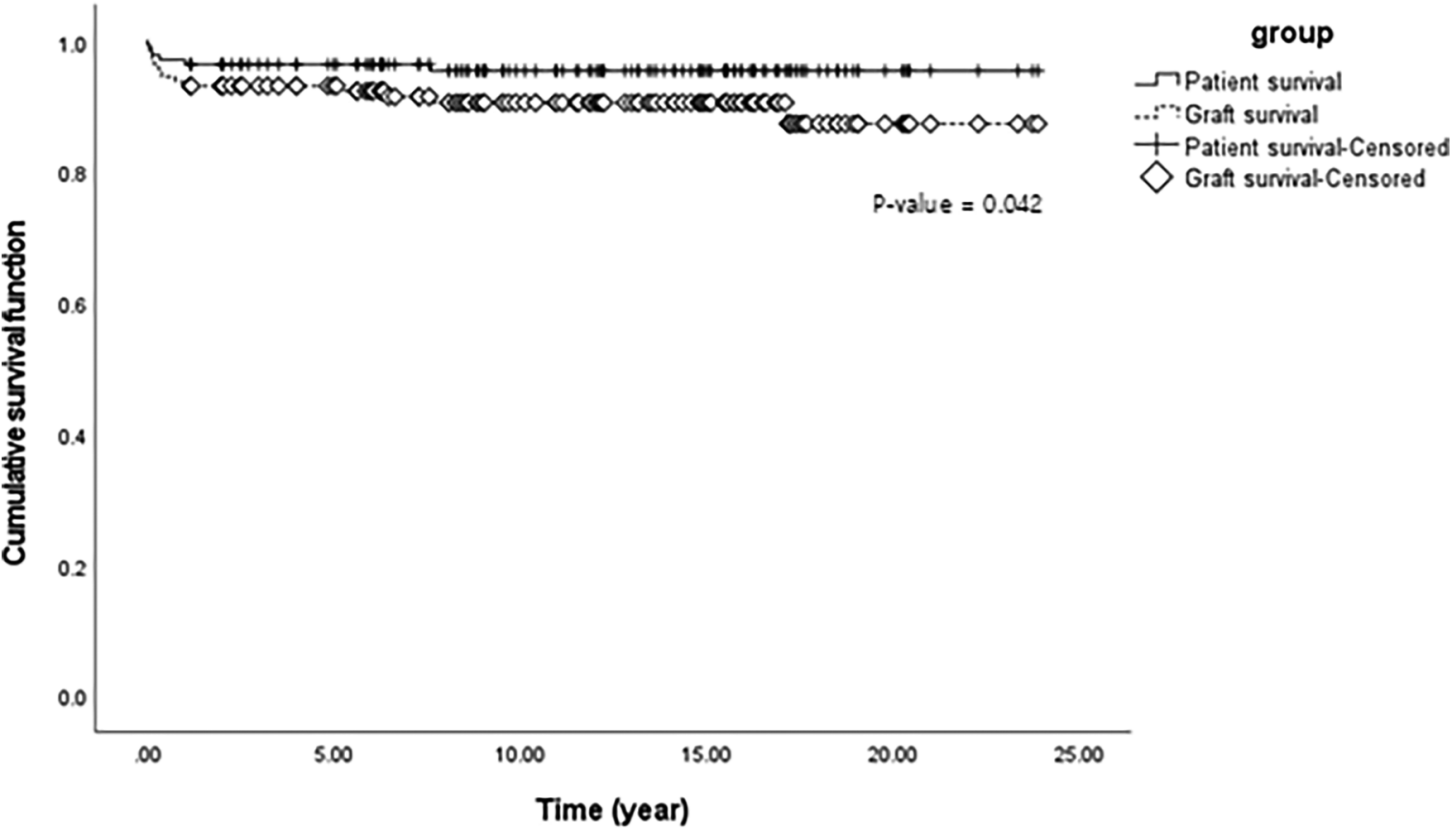
Cumulative patient and graft survival function using Kaplan-Meier survival analysis of liver transplantation in 145 pediatric patients with biliary atresia.

**Figure 2:**
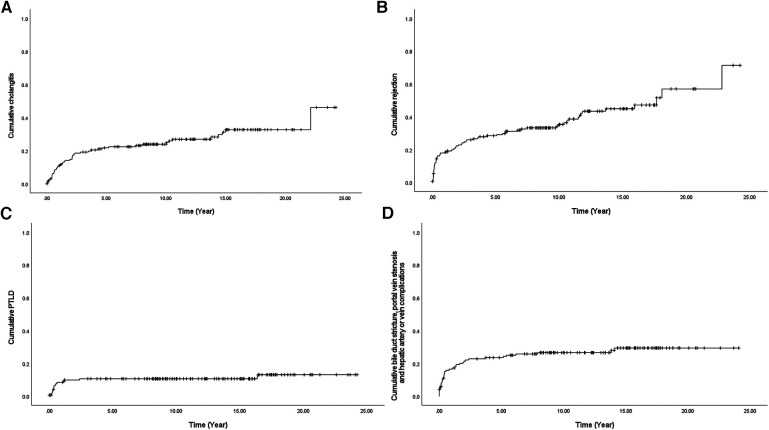
Cumulative complications (**A**) cholangitis, (**B**) rejection, (**C**) PTLD, and (**D**) bile duct stricture, portal vein stenosis, hepatic artery stenosis, or vein problems using Kaplan-Meier survival curve after liver transplantation in 145 pediatric patients with biliary atresia. PTLD, Post-transplantation lymphoproliferative disease.

**Figure 4:**
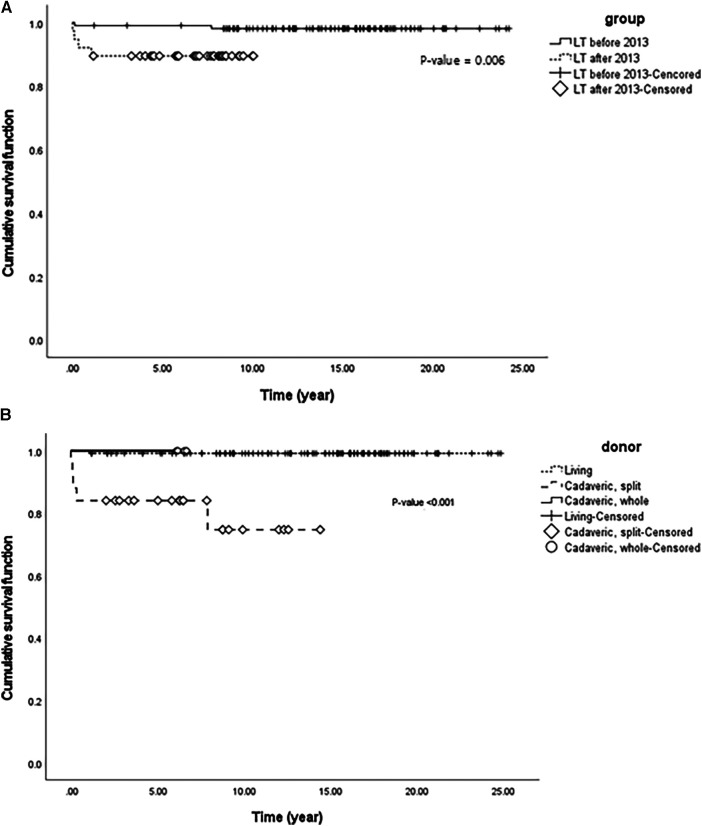
(**A**) Cumulative patient survival function using Kaplan-Meier survival analysis of two groups with liver transplantation classified by the year 2013 in 145 pediatric patients with biliary atresia. (**B**) Cumulative patient survival function using Kaplan-Meier survival analysis of three groups with liver transplantation classified according to donor differences in 145 pediatric patients with biliary atresia.

For descriptive statistics, continuous variables are expressed as mean values with standard deviations and categorical variables as absolute numbers with percentages (Tables 1, 2). Kaplan-Meier survival plots were used to analyze patient and graft survival functions and time-dependent cumulative complications (Figures 1, 2, 4). Univariate and multivariate logistic regression analyses were used to evaluate the risk factors for patient survival, graft survival, and complications (Tables 3, 4). All statistical analyses were performed using SPSS version 27.0 (IBM Corp., Armonk, NY, USA). Statistical significance was set at *p* < 0.05. significant.

**Table 1:** Clinical characteristics, laboratory results, imaging findings before liver transplantation and intraoperative findings of liver transplantation in 145 patients with biliary atresia.

Demographics	*N* = 145
Median age of diagnosis (month)	2.27 (1.70–2.60)
Median age of LT (month)	11.37 (8.37–18.43)
Median time interval to LT (month)	8.83 (6.17–17.5)
Male/Female, *N* (%)	53 (36.6%)/92 (63.4%)
Kasai Op., *N* (%)	131 (90.3%)
Body weight at the time of LT, kg	8.6 (7.3–10.8)
LT donor, *N* (%)	
Living	116 (80%)
Deceased, split	25 (17.2%)
Deceased, whole	4 (2.8%)
PELD score	14.00 (8.75–21.00)
ABO incompatibility, *N* (%)	1 (0.7%)
GRWR (%)	2.90 (2.20–3.70)
Warm ischemic time (min)	29.00 (25.00–33.25)
Total ischemic time (min)	88.00 (73.00–145.00)
Laboratory test	
Total bilirubin (mg/dl)	15.0 (7.8–20.7)
Direct bilirubin (mg/dl)	11.2 (6.2–14.4)
AST (U/L)	214.5 (153.3–305.5)
ALT (U/L)	117.0 (71.5–178.8)
GGT (U/L)	224.5 (125.3–357.5)
INR	1.26 (1.13–1.50)
Ammonia (µmol/L)	78.8 (60.0–112.4)
Albumin (g/dl)	3.2 (2.8–3.6)
Image findings	
Parenchymal change, Y (%)	
Heterogeneous enhancement	105 (72.4%)
Surface nodularity	90 (62.1%)
Fissure widening	61 (42.1%)
Periportal edema	87 (60.0%)
Sign of portal hypertension, Y (%)	
Splenomegaly	136 (93.8%)
Ascites	51 (35.2%)
Gastroesophageal varix	66 (45.5%)
Intraoperative parameters	
Splenomegaly, Y (%)	102 (70.3%)
Hepatomegaly, Y (%)	73 (50.3%)
Cirrhosis, Y (%)	122 (84.1%)
Hepatic vein size (mm)	20.0 (17.0–22.0)
Portal vein size (mm)	14.0 (12.0–15.8)
Bile duct size (mm)	5.0 (4.0–6.0)

Values are expressed as *n* (percentage) or median (interquartile range).

**Table 2:** Clinical outcomes after liver transplantations in the pediatric patients with biliary atresia.

Outcome	*N* = 145
Patient survival, *N* (%)	139 (95.8%)
Median patient survival time (years)	12.33 (8.43–16.48)
Expire after LT, *N* (%)	6 (4.1%)
Post-op. complications	4/6 (66.7%)
Acute rejection	1/6 (16.7%)
Others	1/6 (16.7%)
Median time interval to expire (months)	2.80 (1.31–10.42)
Graft survival, *N* (%)	132 (91.0%)
Median graft survival times (years)	12.10 (6.36–16.25)
Re-liver transplantation, *N* (%)	7 (4.8%)
Median time interval to re-LT (months)	9.23 (5.91–74.5)
Cholangitis	24 (16.6%)
With bile duct stricture	7 (4.8%)
Single event	15/24 (62.5%)
Multiple events	9/24 (37.5%)
Rejection	44 (30.3%)
Single event	31 (70.5%)
Multiple events	13 (29.5%)
Other postoperative complications	
PTLD	16 (11.0%)
Portal vein stenosis	22 (15.2%)
Bile duct stricture	9 (6.2%)
Hepatic artery stenosis	4 (2.8%)
Thrombosis	6 (4.1%)
– Hepatic artery	2
– Portal vein	3
– Hepatic vein	1
Bleeding	3 (2.1%)
Infection	14 (9.7%)
Bowel perforation, anastomosis leakage	6 (4.1%)

Values are expressed as *n* (percentage) or median (interquartile range).

**Table 3:** Risk factors for patient mortality and graft failure after liver transplantation. Multivariate analysis was performed by selecting variables with *p* < 0.1 in univariable analysis.

	Parameter	Multivariate analysis			
		*p*-value	Odds ratio	95% CI	
				Lower	Upper
Patient mortality	Hepatic artery stenosis	**0.006**	119.10	4.03	3,516.46
	Rejection	0.551	3.97	0.04	370.74
	Infection	**0** **.** **010**	9.62	1.71	54.24
	PTLD	0.406	3.16	0.21	47.89
	Age < 1 year	**0** **.** **022**	44.31	1.75	1,124.82
	Donor	**0** **.** **011**	11.29	1.74	73.33
Graft failure	Bile duct stricture	**0** **.** **020**	223.55	6.80	7,346.68
	Multiple cholangitis	**0** **.** **010**	1.174	1.08	1.28
	Rejection	**0** **.** **050**	2.30	0.12	44.26
	Portal vein stenosis	**0** **.** **009**	72.52	2.92	1,799.24
	Donor	**0** **.** **004**	11.12	2.20	56.28
	GRWR	0.052	0.261	0.07	1.01
	PELD	0.429	2.15	0.322	14.37

Bold values mean statistically significant values with *p*-value <0.05.

**Table 4:** Risk factors for complications after liver transplantation. Multivariate analysis was performed by selecting variables with *p* < 0.1 in univariable analysis.

	Parameter	Multivariate analysis			
		*p*-value	Odds ratio	95% CI	
				Lower	Upper
Cholangitis	Age at LT	**0** **.** **049**	1.02	1.00	1.04
	Kasai operation	**0** **.** **025**	0.10	0.01	0.74
	Intraoperative findings				
	(a) Weight to bile duct size	**0** **.** **031**	0.02	0.00	0.703
	(b) Weight to portal vein size	**0** **.** **006**	12.82	2.09	78.84
Bile duct stricture	GRWR	0.074	3.16	0.21	47.89
	Intraoperative findings				
	(a) Weight to bile duct size	**0** **.** **049**	0.03	1.75	1,124.82
	(b) Weight to portal vein size	**0** **.** **015**	16.54	1.72	158.99
Portal vein stenosis	Image finding				
	– Splenomegaly	0.072	3.52	0.90	13.86
	Intraoperative findings				
	– Weight to portal vein size	0.077	0.81	0.64	1.02

Bold values mean statistically significant values with *p*-value <0.05.

## Results

### Pre- and intra-operative findings of LT

Among the 145 pediatric patients, 53 (36.6%) were male and 92 (63.4%) were female (Table 1). The median age of patients at the time of the diagnosis of BA was 2.27 months and the median age at the time of LT was 11.37 months with 8.83 months as the median time interval from diagnosis to LT. The median body weight at the time of LT was 8.6 kg. Most patients (90.3%) underwent the Kasai operation before LT. Most patients (116 cases, 80%) received grafts from living donors. The remaining 29 patients received grafts from deceased donors. In the case of deceased donors, split grafts were used in 25 patients and whole grafts were used in four patients. There were no cases transplanted with reduced size grafts. The median PELD score was 14.00, and one patient underwent non-match ABO blood type LT. The median GRWR was 2.90 and the median warm and total ischemic time were 29.00 min and 88.00 min, respectively.

Characteristically identified pre-operative image findings of parenchymal changes were signs of inflammation (heterogeneous enhancement, 72.4% of patients; periportal edema, 60.0% of patients) and cirrhosis (surface nodularity, 62.1% of patients; fissure widening, 42.1% of patients). Imaging findings suggestive of portal hypertension (splenomegaly, 93.8% of patients; ascites, 35.2% of patients; and gastroesophageal varix, 45.5% of patients) were also observed. When evaluated during the actual operation, there were slight differences in imaging findings. Splenomegaly was confirmed in 70.3% of patients, which was less than that observed in pre-operative imaging tests. Liver cirrhosis was confirmed more frequently (84.1%) during surgery than on imaging. The median hepatic vein size, portal vein size, and bile duct size of the grafts were 20.0 mm (IQR range, 17.0–22.0 mm), 14 mm (IQR range, 12.0–15.8 mm), and 5 mm (IQR range, 4.0–6.0 mm), respectively.

### Clinical outcomes: survival rates and complications

Only six patients died after LT; therefore, the overall patient survival rate in over 20 years was 95.8% (139/145) (Table 2). The median survival time was 12.33 years. Postoperative complications, including pneumonia, sepsis, and bleeding, were the most common causes of death, occurring in four out of six patients. One patient died because of uncontrolled acute rejection, and the other died because of sudden cardiac arrest, the cause of which was unknown. Half of the deaths occurred within 90 days of transplantation indicating that the median survival time from surgery to death was 2.8 months. Seven patients (4.8%) underwent re-LT, and the median time to re-LT was 9.23 months. One patient underwent a third LT due to decreased liver function with chronic liver changes at the final operation. Our results revealed that the overall graft survival rate within over 20 years was 91.0% (132/145). The median graft survival time was 12.10 years which was not significantly different from the median patient survival time. Figure 1 shows the comparison of cumulative patient and graft survival functions over time. Unlike patient survival that decreased mostly at an early stage, graft survival steadily decreased over time (*p* = 0.042).

Cholangitis and rejection were the most frequently observed complications in patients after LT. Cholangitis occurred in 16.6% of the patients. Among them, 37.5% (9/24) experienced multiple episodes of cholangitis. One patient who had up to 24 episodes of recurrent cholangitis, underwent re-LT. A total of 44 patients (30.3%) experienced rejection after LT. Most (70.5%) experienced a single event of rejection; however, 13 patients experienced multiple events of rejection. Portal vein stenosis was observed in 15.2% of patients. Some of the patients underwent multiple ballooning procedures. Other anastomotic or vascular complications included bile duct strictures (4.8%), hepatic vessel stenosis (2.8%), and thrombosis (4.1%—2 cases of hepatic artery, 3 cases of portal vein, and 1 case of hepatic vein). Infections such as pneumonia, sepsis, peritonitis, and wound site infections were observed after LT in 9.7% of patients. There were three cases of massive bleeding that required reoperation to control bleeding. There were also six cases of mechanical problems that occurred during surgery, such as bowel perforation and anastomotic leakage. Other complications included multiple organ failure, wound dehiscence, biloma, pneumothorax, brain edema, and PTLD.

### Steady increase in complications discovered with long-term observation

After a detailed investigation of the high-frequency complications as shown in Table 2, some patterns of complications were observed when all occurrences of cholangitis, rejection, PTLD, and anastomosis site problem of the duct or vessel were counted over a period of 20 years after LT (Figure 2). In the case of PTLD, because EBV is activated as immunity decreases due to the use of IS, PTLD was also frequently observed in the early period of IS use, that is within the first 1–2 years after LT (Figure 2C). In the case of anastomosis site problems of the duct or vessel, it increased steadily within 5 years; however, it was only identified as an intermittent event (Figure 2D). In other words, the possibility of developing a surgical connection problem decreases 5 years after LT. However, in the case of cholangitis and rejection, a steady increase can be seen over a period of more than 20 years, unlike the complications seen above (Figures 2A, B).

### Risk factors associated with patient mortality and graft failure

Table 3 shows the factors associated with patient mortality and graft failure. The factors, age at the time of LT, time interval from BA diagnosis to LT, whether Kasai operation was performed, body weight at LT, type of donor, PELD score, GRWR, and ischemic time, as indicated in Table 1, and all complications listed in Table 2 were included in logistic regression analyses. Regarding cholangitis, only cases with multiple events were included in the analysis. Because the majority of patients with bile duct stricture (7/9, 77.8%) developed multiple cholangitis, multicollinearity problems may occur, so statistical analysis was performed including only the bile duct stricture or multiple cholangitis with other factors. Table 3 is the result of performing logistic regression including both factors after confirming the intercorrelation was not large. In the multivariate analysis, hepatic artery stenosis, infection, age <1 year at LT, and deceased split graft of donor type were evaluated as risk factors affecting patient mortality, with *p* values of 0.006, 0.010, 0.022, and 0.011, respectively. The factor with the highest odds ratio was hepatic artery stenosis with an odds ratio of 119.10, followed by age <1 year at LT, deceased split graft of donor type, and infection with odds ratios of 44.31, 11.29, and 9.62, respectively. Factors affecting graft failure were evaluated using the same factors as those used in the analysis of patient mortality. In the multivariate analysis, bile duct stricture, multiple cholangitis, rejection, portal vein stenosis, and deceased split graft of the donor type were evaluated as risk factors for graft failure, with *p*-values of 0.020, 0.010, 0.050, 0.009, and 0.004, respectively. The factor with the greatest odds ratio was bile duct stricture (223.55), followed by portal vein stenosis, deceased split graft of donor type, rejection, and multiple cholangitis with odds ratios of 72.52, 11.12, 2.30, and 1.18, respectively.

### Risk factors associated with the complications affecting graft failure

Table 4 shows the factors that affect the complications (cholangitis, bile duct stricture, and portal vein stenosis) associated with graft failure as mentioned in Table 3. Demographic characteristics (age at the time of BA diagnosis, age at the time of LT, time interval from BA diagnosis to LT, whether the Kasai operation was performed, body weight at LT, type of donor, PELD score, GRWR, and ischemic time), imaging findings, and intraoperative parameters, as indicated in Table 1, were included in logistic regression analyses. For intraoperative parameters, sizes were divided by body weight for analysis. In the multivariate analysis, age <1 year at LT and weight to portal vein size were evaluated as risk factors that affect cholangitis, with *p* values of 0.049 and 0.006, respectively, and odds ratios of 1.01 and 12.82, respectively. The protective factors for preventing cholangitis were Kasai operation and weight to bile duct size, with *p*-values of 0.025 and 0.031 and odds ratios of 0.010 and 0.02, respectively. In the multivariate analysis related to the bile duct stricture, weight-to-bile duct size was evaluated as a protective factor and weight-to-portal vein size as a risk factor, as in the evaluation of cholangitis (odds ratio = 0.03, *p* = 0.049 and odds ratio = 16.54, *p* = 0.015, respectively). No significant factor was found in the case of portal vein stenosis.

### Results of policy changes for LT

**Figure 3:**
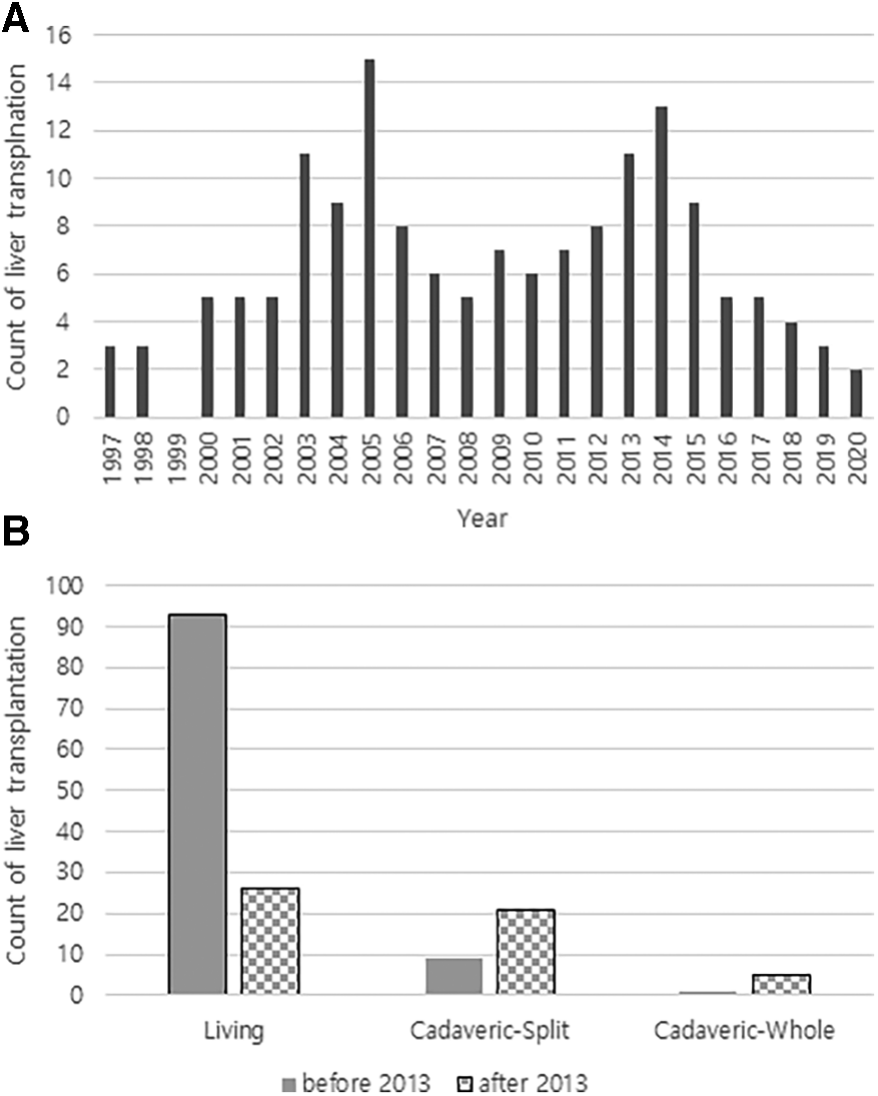
(**A**) Annual number of liver transplantations at a single tertiary referral center in South Korea. (**B**) Counts of liver transplantations according to donor type before and after 2013.

Since the policy on the indication of split liver grafts of deceased donor was expanded in Korea, the total number of LT and the number of cases of LT with split graft increased (Figure 3). We analyzed patient survival over time by comparing the two groups classified by the number of LT before 2013 and that after 2013 (Figure 4A). The group that underwent LT after 2013 showed significantly lower survival over time than the group that underwent LT before 2013 (*p* = 0.006). Based on this result, when the survival was evaluated according to the donor type regardless of the specific time period, the survival over time of the split graft of the deceased donor was significantly lower than that of the other two donor types (*p* < 0.001; Figure 4B).

## Discussion

We retrospectively reviewed the clinical outcomes of 145 pediatric patients who underwent LT for BA. This study included LT patients of young age, mostly in infants, and observed the patients for more than 20 years. We attempted to evaluate the major factors that cause the transplanted liver to develop chronic liver disease through long-term observations. This is because graft survival is directly associated with the quality of life of patients and can be improved if patients are managed more systematically to prevent progression to chronic liver disease.

One of our major findings was the time difference in the onset of complications. The complications that were frequently observed were PTLD, rejection, cholangitis, and anastomosis site problems of the vessels or ducts. Several previous studies have also investigated complications after LT, but most of them have focused on the early phase of complications or on the long-term observation of one specific complication (10–14). Regarding PTLD, because it is associated with immunity, its risk starts to increase with the initiation of treatment with IS; the incidences were primarily in the early observation period, within the first 1–2 years after LT. Regarding mechanical problems of the anastomosis site of the graft, their risk increased steadily within 5 years and was rarely observed after 5 years. Unlike the above-mentioned complications, cholangitis and rejection rates have steadily increased over the last 20 years. In addition, multiple events such as cholangitis, rejection, and portal vein stenosis (a part of the anastomosis site problem) were evaluated as risk factors for graft failure in the multivariate analysis. Hence, repeated cholangitis and rejection eventually leads to the progression of the transplanted liver to chronic liver disease that requires re-LT.

Understanding the existence of time differences in complications allow clinicians to focus on and devise treatment strategies for each period. Based on the results of this study, it is recommended that clinicians monitor IS concentrations and regularly evaluate EBV titers for up to 1–2 years after transplantation. In the case of rejection, acute rejection is well known for its pathophysiology and treatments; however, chronic rejection is not. Acute rejection is followed by chronic rejection, and it can occur steadily for 20 years. Therefore, clinicians should be aware of treatments for chronic rejection. As a treatment for chronic rejection, mTOR inhibitors, such as sirolimus and everolimus, can be added to CI when the liver injury is irreversible, along with an escalation of the dose of IS (15). Clinicians should also monitor the concentration of CI to ensure that it is within the therapeutic range, and also monitor patients’ compliance with drug intake. Regarding the management of cholangitis, prophylactic medications with sulfamethoxazole/trimethoprim and phenobarbital for the prevention of cholangitis are routinely administered after the Kasai operation, but not after LT. Since the cumulative risk of cholangitis steadily increases in over 20 years, we suggest continued administration of prophylactic antibiotics as after Kasai operation. This requires prospective studies to evaluate definitive differences in the incidence of cholangitis. Unlike many studies on prophylactic antibiotic administration after Kasai operation, there are currently no papers evaluating the administration of prophylactic antibiotics after LT.

Our second finding is that the weight-to-duct or vessel size is a more important factor to be considered, in terms of complications, than the GRWR. In this study, the analysis of risk factors that affect multiple events of cholangitis and bile duct stricture commonly revealed that a larger weight to bile duct size ratio was a protective factor against complications; however, a larger weight to portal vein size ratio was a risk factor for complications (Table 4). Regarding size, particularly in LT in patients with BA that was performed in the neonatal period, large GRWRs sometimes induce large-for-size graft syndrome because of limited number of pediatric donor livers leads to the use of adult livers (16, 17). This size mismatch usually requires more complex portal vein anastomoses because of the different calibers between the donor and recipient vessels, as well as inadequate portal vein length. These complexities can cause posttransplant complications, such as portal flow anomalies, stenosis of the portal vein anastomosis, and portal vein thrombosis (18). These factors can lead to revascularization failure after transplantation. In this study, patients who failed revascularization had a larger average weight to portal vein size compared to patients who did not fail (Supplementary Table S1). This means that the larger the size mismatch, the more likely revascularization will fail. Pediatric patients who received an adult liver have a basic-size mismatch due to a larger portal vein from the adult donor; furthermore, a larger weight-to-portal vein size means an increased size mismatch. A larger portal vein size of the donor can be a risk factor for complications. The larger bile duct drains bile adequately and the possibility of narrowing of the duct is low; therefore, the weight to bile duct size ratio appears to have a protective effect, as predicted. Contrary to the author's expectations, vessel and duct sizes were evaluated as being more meaningful than the GRWR. This suggests that the vessel and duct sizes are more suitable for evaluating the flow and drainage.

Organ transplantation has been highly influenced by the policies. Most of the changes in organ transplant policies have gone in the good direction of increasing the number of transplants and reducing waitlist mortality (19–21); however, some policy changes have caused unexpected side effects. Trivedi et al. (22) found that policy change significantly increased the number of patients listed and transplanted with acute mechanical circulatory support, resulting in a reduced waiting time for transplanted patients while increasing the distance traveled to procure a donor heart, donor ischemic time, and early post-transplant mortality. In Korea, the number of eligible donors is greater than the number of recipients. However, due to their small body size, it is often necessary for children to receive a split graft rather than a whole graft from a deceased donor. To reduce this supply-demand mismatch and the number of recipients on the transplant waiting list, the indications for split grafts from deceased donors were expanded in 2013. The revised content expanded the recipient indications by removing the rule that their parents must explain why they are unsuitable liver donors. This change in policy caused a change in the donor-type ratio in LT in Korea (7). Our third finding is that survival outcomes have changed because of policy changes that affect the donor-type ratio in transplantation. Split grafts are generally known to have a poorer prognosis than living donors or whole grafts from deceased donors because of the longer ischemic time and genetic irrelevances (23–26). Although it was not possible to accurately determine the number of patients who died on the waiting list, it was confirmed that cumulative survival decreased after 2013, when the policy was changed.

This study had some inherent limitations because of its retrospective, single-center design. First, as the follow-up period was long, not one but two surgeons performed LT, which may have affected complication outcomes. However, the surgeon did not change suddenly, there was an overlap period of approximately 6 years. Second, as the data were collected by evaluating EMR records, the frequency of complications may have been underestimated.

## Conclusion

As a long-term follow-up study, this study revealed that there were time differences in complications after LT in children with BA. Understanding the existence of time differences in complications will allow clinicians to focus on and devise treatment strategies for each period to reduce graft failure and increase the quality of life of patients. Evaluation of the weight-to- duct or vessel size is a more important factor to be considered for complications than the GRWR. This result supports recent studies that have asserted the importance of portal flow, rather than the GRWR, in relation to the prognosis of LT. Survival outcomes can be altered by a policy change that affects the donor type ratio in transplantation, although this policy has reduced the number of patients on the waitlist. Since it is a good policy in that it reduces deaths that occur while waiting for a transplant, it is necessary to comprehensively analyze the number of deaths on the waiting list and the number of survivals after transplant before and after the policy change using national data. As a result, policies are as important as changes in surgical techniques or medications in transplantation.

## Data Availability

The original contributions presented in the study are included in the article/**Supplementary Material**, further inquiries can be directed to the corresponding authors.

## References

[1] Lee KJ Kim JW Moon JS Ko JS. Epidemiology of biliary atresia in Korea. J Korean Med Sci. (2017) 32:656–60. 10.3346/jkms.2017.32.4.656 28244293 PMC5334165

[2] Shen W-J Chen G Wang M Zheng S. Liver fibrosis in biliary atresia. World J Pediatr. (2019) 15:117–23. 10.1007/s12519-018-0203-1 30465125

[3] Lee S Park H Moon S-B Jung S-M Kim JM Kwon CHD Long-term results of biliary atresia in the era of liver transplantation. Pediatr Surg Int. (2013) 29:1297–301. 10.1007/s00383-013-3366-9 23948814

[4] Moreno R Berenguer M. Post-liver transplantation medical complications. Ann Hepatol. (2006) 5:77–85. 10.1016/S1665-2681(19)32022-8 16807513

[5] Pascher A Neuhaus P. Bile duct complications after liver transplantation. Transpl Int. (2005) 18:627–42. 10.1111/j.1432-2277.2005.00123.x 15910286

[6] Haddad L Cassenote AJF Andraus W de Martino RB Ortega N Abe JM Factors associated with mortality and graft failure in liver transplants: a hierarchical approach. PLoS One. (2015) 10:e0134874. 10.1371/journal.pone.0134874 26274497 PMC4537224

[7] Yoon KC Song S Lee S Kim O-K Hong SK Yi N-J Outcomes of split liver transplantation vs living donor liver transplantation in pediatric patients: a 5-year follow-up study in Korea. Ann Transplant. (2022) 27:e935682–1. 10.12659/AOT.935682 35502129 PMC9084422

[8] Diamond IR Fecteau A Millis JM Losanoff JE Ng V Anand R Impact of graft type on outcome in pediatric liver transplantation: a report from Studies of Pediatric Liver Transplantation (SPLIT). Ann Surg. (2007) 246:301–10. 10.1097/SLA.0b013e3180caa415 17667510 PMC1933573

[9] Roberts JP Hulbert-Shearon TE Merion RM Wolfe RA Port FK. Influence of graft type on outcomes after pediatric liver transplantation. Am J Transplant. (2004) 4:373–7. 10.1111/j.1600-6143.2004.00359.x 14961989

[10] Taylor SA Venkat V Arnon R Gopalareddy VV Rosenthal P Erinjeri J Improved outcomes for liver transplantation in patients with biliary atresia since pediatric end-stage liver disease implementation: analysis of the society of pediatric liver transplantation registry. J Pediatr. (2020) 219:89–97. 10.1016/j.jpeds.2019.12.023 32005543

[11] Sieders E Peeters PM TenVergert EM de Jong KP Porte RJ Zwaveling JH Early vascular complications after pediatric liver transplantation. Liver Transpl. (2000) 6:326–32. 10.1053/lv.2000.6146 10827234

[12] Stringer MD Marshall MM Muiesan P Karani JB Kane PA Mieli-Vergani G Survival and outcome after hepatic artery thrombosis complicating pediatric liver transplantation. J Pediatr Surg. (2001) 36:888–91. 10.1053/jpsu.2001.23963 11381419

[13] Buell JF Funaki B Cronin DC Yoshida A Perlman MK Lorenz J Long-term venous complications after full-size and segmental pediatric liver transplantation. Ann Surg. (2002) 236:658. 10.1097/00000658-200211000-00017 12409673 PMC1422625

[14] Laurence JM Sapisochin G DeAngelis M Seal JB Miserachs MM Marquez M Biliary complications in pediatric liver transplantation: incidence and management over a decade. Liver Transpl. (2015) 21:1082–90. 10.1002/lt.24180 25991054

[15] Choudhary NS Saigal S Bansal RK Saraf N Gautam D Soin AS. Acute and chronic rejection after liver transplantation: what a clinician needs to know. J Clin Exp Hepatol. (2017) 7:358–66. 10.1016/j.jceh.2017.10.003 29234201 PMC5715482

[16] Tanaka K Ogura Y. Small-for-size graft” and” small-for-size syndrome” in living donor liver transplantation. Yonsei Med J. (2004) 45:1089–94. 10.3349/ymj.2004.45.6.1089 15627301

[17] Goldaracena N Echeverri J Kehar M DeAngelis M Jones N Ling S Pediatric living donor liver transplantation with large-for-size left lateral segment grafts. Am J Transplant. (2020) 20:504–12. 10.1111/ajt.15609 31550068

[18] Alvarez F. Portal vein complications after pediatric liver transplantation. Curr Gastroenterol Rep. (2012) 14:270–4. 10.1007/s11894-012-0257-5 22434261

[19] Ishaque T Massie AB Bowring MG Haugen CE Ruck JM Halpern SE Liver transplantation and waitlist mortality for HCC and non-HCC candidates following the 2015 HCC exception policy change. Am J Transplant. (2019) 19:564–72. 10.1111/ajt.15144 30312530 PMC6349527

[20] Chopra B Sureshkumar KK. Changing organ allocation policy for kidney transplantation in the United States. World J Transplant. (2015) 5:38. 10.5500/wjt.v5.i2.38 26131405 PMC4478598

[21] Kalra A Wedd JP Biggins SW. Changing prioritization for transplantation: MELD-Na, hepatocellular carcinoma exceptions, and more. Curr Opin Organ Transplant. (2016) 21:120–6. 10.1097/MOT.0000000000000281 26825358

[22] Trivedi JR Slaughter MS. “Unintended” consequences of changes in heart transplant allocation policy: impact on practice patterns. ASAIO J. (2020) 66(2):125–7. 10.1097/MAT.0000000000001128 31977354

[23] Kwong A Kim W Lake J Smith J Schladt D Skeans M OPTN/SRTR 2018 annual data report: liver. Am J Transplant. (2020) 20:193–299. 10.1111/ajt.15674 31898413

[24] Farmer DG Venick RS McDiarmid SV Ghobrial RM Gordon SA Yersiz H Predictors of outcomes after pediatric liver transplantation: an analysis of more than 800 cases performed at a single institution. J Am Coll Surg. (2007) 204:904–14. 10.1016/j.jamcollsurg.2007.01.061 17481508

[25] D’alessandro A Knechtle S Chin LT Fernandez L Yagci G Leverson G Liver transplantation in pediatric patients: twenty years of experience at the university of Wisconsin. Pediatr Transplant. (2007) 11:661–70. 10.1111/j.1399-3046.2007.00737.x 17663691

[26] Mogul DB Luo X Bowring MG Chow EK Massie AB Schwarz KB Fifteen-year trends in pediatric liver transplants: split, whole deceased, and living donor grafts. J Pediatr. (2018) 196:148–53.e2. 10.1016/j.jpeds.2017.11.015 29307689 PMC5924625

