# Supplementary material for: Long-term outcomes of liver transplantation for biliary atresia and results of policy changes: over 20 years of follow-up experience
Source: Front Pediatr. 2024 Mar 1;11:1242009. doi: 10.3389/fped.2023.1242009 (PMC10940458; doi:10.3389/fped.2023.1242009)
Supplement: Supplementary file 1 [file Table1.docx]

|  | **Revascularization** | | **P value** |
| --- | --- | --- | --- |
|  | **Fail group (n=28)** | **Non-fail group (n=117)** |  |
| Weight to portal vein size | 1.52 ± 0.66 | 1.27 ± 0.65 | 0.076 |

Supplementary table 1. Comparison of weight to portal vein size between the group without revascularization failure and the group without revascularization failure
